# Data sharing policy design for consortia: challenges for sustainability

**DOI:** 10.1186/gm523

**Published:** 2014-01-29

**Authors:** Jane Kaye, Naomi Hawkins

**Affiliations:** 1 HeLEX - Centre for Health, Law and Emerging Technologies, Department of Public Health, University of Oxford, Old Road Campus, Oxford OX3 7LF, UK; 2 University of Exeter Law School, Amory Building, Rennes Drive, Exeter EX4 4RJ, UK

## Abstract

The field of human genomics has led advances in the sharing of data with a view to facilitating translation of research into innovations for human health. This change in scientific practice has been implemented through new policy developed by many principal investigators, project managers and funders, which has ultimately led to new forms of practice and innovative governance models for data sharing. Here, we examine the development of the governance of data sharing in genomics, and explore some of the key challenges associated with the design and implementation of these policies. We examine how the incremental nature of policy design, the perennial problem of consent, the gridlock caused by multiple and overlapping access systems, the administrative burden and the problems with incentives and acknowledgment all have an impact on the potential for data sharing to be maximized. We conclude by proposing ways in which the scientific community can address these problems, to improve the sustainability of data sharing into the future.

## Introduction

Genomics research has led the scientific community in implementing the principle of open access to enable wide-scale sharing of data derived from human beings [1]. A number of key documents relating specifically to genomics research have established the principle of open access [2-4], which has affected every aspect of genomic science, from recruitment of research participants through to the publishing of research results. The rationale behind these policies is that research results and data generated through public funding should be freely available to the wider research community, to realize their full benefit. The National Institutes of Health (NIH) considers that data sharing “is essential to facilitate the translation of research results into knowledge, products, and procedures that improve human health” [5]. To implement these data sharing principles, new policy has been developed by many principal investigators, project managers and funders, which has ultimately led to new forms of practice and innovative governance models for data sharing, from projects such as the Wellcome Trust Case Control Consortium (WTCCC) to the innovative Personal Genome Project, which uses the principle of open consent [6-8].

As data sharing in genomics research involving human subjects has been carried out now for a number of years, reconsideration of the issues arising from design and implementation of data access policies is timely and appropriate. The NIH has recognized the need to revise and update its data sharing policy [2]. Groups such as the Global Alliance are exploring how to enable data sharing through various initiatives, including better harmonization, creation of standards and development of incentives [2,3]. Here, we explore some of the important issues associated with the design and implementation of data sharing policies. In particular, we focus on the processes used by scientists and some of the key challenges that are associated with the development and implementation of data access policies. We examine the implications of existing policy design mechanisms, which often involve incremental approaches that build on previous best practice, for the long-term sustainability of data sharing of genomics research involving human subjects, and we propose ways in which policy design can be streamlined and improved to maximize the potential benefits of data sharing for the genomics and biomedical research communities.

## The history of data access policies

The Human Genome Project (HGP) was a significant moment in the history of human genomics, because it endorsed the principle that all sequencing data would be deposited on the web to be immediately accessible by the research community [4]. This decision built on the success of practices that had been established in a number of other molecular biology projects such as the PDB database for protein structures [9] and EMBL/GenBank/DDBJ for DNA sequences [10]. The *C. elegans* genome project [11] established important precedents in the coordination and organization of specialist teams focused on the sequencing of one organism. The first policy in human genomics for pre-publication sequence data was described in the Bermuda Principles (1996), which embodied the open access approach taken in the HGP [12]. This was followed by the Fort Lauderdale Agreement in 2003 [13] and the Toronto Statement in 2009 [14], both of which further endorsed the principle of rapid data release through the web for sequence data. These principles were applied in other projects that were established to set up sequence reference databases such as the HapMap Project (2002–2009) [15] and the 1000 Genomes Project (2008–2012) [16].

After the HGP, new research approaches, such as genome-wide association studies (GWAS) were used to further understand the influence of genomes on disease. To facilitate this approach, projects were established with the support of funders to generate and compare the sequence data drawn from samples collected by clinically based researchers, usually as part of specific disease studies. Examples of such projects are the WTCCC [7], Genetic Association Information Network [17] and Database of Genotypes and Phenotypes (dbGaP) [18], which were data-generating projects as well as providing lasting community resources. As part of these projects, new governance structures were developed so that the rest of the research community could access the GWAS sequence data and phenotypic data that had been generated by specific principal investigators (PIs). Data access committees (DACs) were established to manage access requests but also to consult and defer to PIs if necessary. The aim of this ‘managed access’ model was to protect the interests of the participants who had donated the samples as well as the PIs, who were responsible for the recruitment of patients and were the custodians of the samples. This was a very different model from the one that had been developed for the HGP and subsequent GWAS data-generating projects.

One of the key lessons learnt from the GWAS initiatives was that the open access web approach to sequence data that had been a feature of the HGP was no longer feasible if the confidentiality commitments that had been made to research participants were to be upheld. Many of the GWAS repositories had placed summary sequence data on the web so that researchers could see this data before applying through the managed access system to access additional datasets or to collaborate with PIs. However, when it was demonstrated that individuals could be distinguished from aggregated single nucleotide polymorphism data [19], the freely available summary GWAS data was withdrawn and placed under managed access controls. This has resulted in researchers now having to apply to access all data through the managed access system, which requires a formal application stating the researchers’ credentials and research proposal followed by approval from the DAC. Concerns about the privacy risks for participants and the linkage of sequence data to phenotypic data have meant that ‘managed access’ repositories have become the norm, as they allow data to be released to other researchers while maintaining responsible scientific practice [20]. This is now the most common form of governance for projects and large consortia.

A significant feature of data access policies is that they have been developed with the broad participation of many stakeholders in the research community. Since the HGP, the culture of openness has resulted in policies being developed for opening up other health datasets for public health purposes [21] and making publications open access [22]. Central repositories have been established for sequence data such as the US dbGaP [23] and the European Genome-phenome Archive (EGA) [24]. By starting to make data sharing a condition of funding support, but also providing repositories for the deposition of data, funders have worked with the scientific community to further the open access agenda [25]. One of the challenges has been how to interpret the broad aspirations found in policy documents into practical governance mechanisms that can guide day-to-day scientific research. As Pryor [26] observes about funders’ policies in the UK, “unfortunately, none of them provides explicit guidance in this matter, although generally they acknowledge that different approaches to data sharing will be required in different situations, making it appropriate for researchers to determine their own strategies for data sharing.” A common approach of funders’ policies is not to prescribe how data should be shared, but rather require that researchers have a plan for doing so [27]. Such an approach respects the expertise of PIs and the diversity of practice that exists across different scientific fields but places considerable responsibility on researchers to develop a suitable governance structure to enable the sharing of data.

## Policy design - incremental policy development

One of the challenges in implementing an open access policy is identifying the governance structures and procedures that are needed. For many researchers, the first step is examining the policies of similar projects or consulting with leaders in the field who have designed or implemented data sharing systems [28]. Several repositories have led the field in designing policies for purpose-built managed access databases, such as dbGaP and the EGA. Large projects have also provided templates for consortia, such as the WTCCC [29], the International Cancer Genome Consortium [30] and MalariaGEN [31]. All of these projects have had a dedicated team of ethicists, lawyers and people experienced in policy development to focus on the development of the data access elements of the project. A feature of these governance systems is the establishment of DACs, which are pivotal in assessing applications to access datasets.

The governance structures of these first projects have become the template for the projects and large consortia that follow. There are both benefits and drawbacks to this incremental approach that builds on previous best practice. Using previous protocols or established methodologies saves time because there is already a tried and tested proof of principle. In addition, these policies have a stamp of approval and a certain amount of credibility as they have often been reviewed by an external body, such as a research ethics committee. An important advantage of using such ‘off-the-peg’ governance systems is that they provide a practical and efficient solution for scientists under time pressures to design a workable process. It allows rapid dissemination of practical experience and permits the diffusion of the effective elements of policies. However, off-the-peg governance systems are not necessarily appropriate for all situations. This is because some projects can require specific solutions, with generic clauses not fitting with the circumstances of all projects. For example, if a project includes samples collected from participants in the developing world, an off-the-peg governance system developed in the USA or Europe will not include the specific provisions that will probably be necessary to accommodate differing models of consent, or that provide appropriate protection for participants facing different risks [32].

A further concern is that the process described above can result in an uncritical adoption of existing practice in the field, rather than appropriate deliberation as to what should be best practice for the field as a whole. Although it is perfectly understandable that policy is designed in this way, the dangers are that the policies, procedures and practices that develop may not always be coherent when they are put together as part of a broader meta-governance. To facilitate global data sharing, funders and other stakeholders need to move from concern for national priorities to the development of a global data sharing and access system that is streamlined and coordinated. Such a structure could expedite access to a number of similar datasets at once wherever they were located in the world. A challenge of such a system would be ensuring that custodians of datasets were consulted about potential uses of the data.

## Access requirements

Currently, governance structures and the requirements for access to data are designed around projects rather than the type of data, which means that the same governance structures are being applied to all data. Having the same system of approval for all datasets does not recognize that the risks of disclosure or harm will vary with different datasets. Some data may have lower risks of identifiability for the participants involved, and may be less sensitive. For example, projects that could potentially result in the identification of individuals and allow the inference of information about sexual health might require higher levels of scrutiny than other projects. Having differing levels of DAC oversight proportionate to risks involved in using some datasets may be more appropriate.

Although it may be important that the specific requirements of individual projects be managed through access regimes designed to fit those projects, when the access systems for individual projects are put together, they may have the cumulative effect of slowing down access to data. The requirement for separate applications to individual projects each time data is needed not only causes delay and involves additional costs for research activities but is also an obstacle to the long-term sustainability of data sharing. If data users need to access data from a large number of projects and there are separate systems of access for each of these projects, with slightly different requirements and forms for each, data users will be faced with an effective gridlock. For example, the criteria on which an application is assessed by a DAC can vary between projects, but also between jurisdictions. In cases in which multiple datasets are needed, only some researchers will have the means to access the data required to carry out the highest quality research, because of the significant burdens in terms of administrative effort of data access applications.

Considering that the purpose of data sharing is to maximize data usage [33], this potential gridlock problem constitutes a significant obstacle to sustainability. It could be addressed proactively through better triaging of the risks associated with using different types of data, to minimize the burdens of access (particularly for aggregated data), and ensuring global, coordinated systems of access. Institutional agreements that cover all access requests to a dataset by all researchers in an institution are needed to supplement oversight by DACs. Such institutional agreements are common with digital library resources and archives and, although the two types of resources are quite different, the agreements may provide a model for systems of access. The research community needs to work towards global meta-governance solutions to cut down on bureaucracy and to support research that requires data from a number of sources.

## Consent

The lack of appropriate consent from research participants has sometimes been a barrier to sharing data. This is because obtaining consent prior to research participation is a fundamental requirement for ethical and lawful research practice. Informed consent is therefore one of the major protections in place for participants and has become the framework that determines whether data on human subjects can be distributed and accessed for further research. The signed informed consent form has become the record of what participants have agreed to, and if this does not mention that data will be shared with other researchers, then best practice requires that this should be respected and data sharing will not be possible. Until recently, many projects did not ask for consent for data sharing, unless they were projects such as the HapMap Project and the 1000 Genomes Project, whose consent forms stipulated that all data would be openly distributed on the internet. The difficulty has been for projects that have been carried out at a time when wide-scale data sharing was not envisaged.

In such cases, it is usually the researchers who make the initial judgment as to whether the scope of the consent covers data sharing and whether the matter should be referred to another decision maker for consideration. This dilemma can place researchers in a difficult position, as they have drafted the consent form in the first place, and it would be tempting to interpret the scope of the consent rather broadly, especially as contacting participants to gain additional consent (‘re-consenting’) can be a costly and time-consuming task that may also inconvenience research participants unnecessarily. With the current paper-based consent process, which is locked in time at the beginning of the research process, it is very difficult to ‘future-proof’ consent forms to cover every eventuality. As a solution, a broad consent to data sharing has often been obtained, which leads to the invidious situation that research participants are not informed about all the uses of their data because this is impossible at the time of recruitment. Moreover, such broad consent is contrary to privacy and data protection principles developed in many countries. To address these concerns, new online dynamic consent communication portals are being developed to enable research participants to receive information about research and to give consent to the use of their personal information for different purposes as it moves through networks [34-36].

If consent forms do not explicitly mention data sharing, referral is usually made to independent review boards, research ethics committees, DACs or in some cases a patient group for deliberation. In such situations, decision-making committees are heavily dependent on the wording of the consent form. Deliberations can become formalistic as the context of consent is necessarily stripped away, and whether data sharing is permitted under the original consent is construed as a black/white, yes/no issue. Some committees may use a more inclusive view of respect for consent, which may pay some attention to the ‘spirit’ of the consent, often as interpreted by the original PI. The risk of such an approach is that it may be too loose when there is a strong impetus to use the data in a way that does not respect the wording of the consent. For example, when participants gave consent to research only for a particular disease, use of their samples as controls for research into another unrelated disease is use that is inconsistent with a formal interpretation of the original consent. However, this might be seen to be consistent with the ‘spirit’ of consent, or justified by the need for samples for this type of research. Although it is not a use that is consistent with the formal wording of the original consent of the participants, it may be in conformity with the views of participants if they were asked.

Although there are differences in approach between jurisdictions and committees, common factors are that such deliberations can require considerable time, and decision-making is placed in the hands of experts rather than in the hands of the research participants themselves. Until a transition is made to electronic forms of governance, the paper informed consent form remains the primary record of what participants have consented to and re-consenting will continue to be costly and time-consuming. The benefits of the dynamic consent system is that it allows multiple different consents to be presented to participants through an online portal, ranging from a broad consent for specific classes of research to an explicit consent for clinical trials. Such a system provides an efficient way to re-contact individuals and to communicate and engage with them, so that they can make decisions about the use of their data in real time. The dynamic consent model provides the means to obtain consent for the use of data as it is shared in different research networks in an ethical and lawful manner.

## New forms of acknowledgement

When designing new governance systems for sharing data there are various considerations that policy makers and researchers need to take into account (Box 1). Protecting the interests of participants has always been the foremost concern of researchers, regulators and policy makers, but the desire to share data and the need to protect privacy have often been characterized as two binary opposites, with privacy being a barrier to sharing. This has tended to obscure other concerns. It has become apparent that there are other areas of tension, such as appropriate recognition of data generators [6] and how to ensure fair access to resources [37], which are outside the traditional domain of research ethics but are just as important to the success of data sharing. To deal with these issues, new procedures and governance structures have been developed to acknowledge and reward essential activities.

Currently, career metrics, which affect hiring, tenure and assessments of research productivity, are orientated around publications rather than the creation of datasets for use by other researchers [38]. Although funders consider that data sharing increases the benefits that have been made to the public in research [39] and researchers can see the benefit of sharing some kinds of data [40], there are also disadvantages to implementing data sharing policies. The following quotes illustrate this: “disincentives to sharing research data include lack of reward or credit for sharing, the substantial amount of labor required to document data in reusable forms, concerns for misuse or misinterpretation of data, control over intellectual property, and the need to restrict access or to de-identify data on human subjects” [41]; “researchers may feel that it is not worth collecting the data in the first place, and that an easier path to publication, and scientific glory, is simply to regularly request access to data that colleagues have collected” [40]. As a way to deal with these concerns, some DACs impose publication moratoriums that give data generators a 6-month lead time to publish from the data before other researchers can have access to it. Such moratoria can also be enforced by publications committees and breaches have been dealt with effectively [42,43]. As well as publication moratoria, technological identifiers (IDs), such as Bioresource Research Impact Factor for biobanks [44] and the Open Researcher and Contributor ID [45], are forms of accreditation and acknowledgement schemes that provide a means of allocating credit for data generation in novel ways. These schemes help but do not in themselves completely address the problem. The genomics community needs to continue to develop novel means of allocating credit so that data generators and data users can be appropriately rewarded and incentivized.

## Administrative burden

There is a broad consensus in the scientific community that there is a need for managed access to most types of genomic data [39]. However, deciding whether to enable access to datasets involves deliberation and decision-making. A significant concern for those responsible for managing projects is the additional administrative load this creates. The process of designing policies, implementing them and administering applications for access are all considerable burdens. This can be disproportionately onerous for smaller and less well funded institutions and projects [41]. Often, management of access falls to a small number of people in a consortium who have the appropriate skills. For many project managers in consortia where large datasets are managed, data access can become a full time occupation. These costs are largely hidden, but greater attention needs to be given to the ongoing costs associated with managing data sharing activities and ideally this needs to be factored into grant applications.

Another concern is that the current administrative oversight of managed access systems based on DACs may be undermining the principle of open access. As a result of open access policies, many genomic datasets are firstly managed by project DACs and when the project ends they are deposited in the managed access archives such as dbGaP and EGA. Despite the huge investment in developing these archives, the data deposited in them is accessed and used relatively rarely compared with data deposited for use on the internet, such as in the HapMap project [46,47]. Often, a system of triaging is used to cut down on the applications requiring review by a DAC. But despite this, a large proportion of the assessment is carried out by these committees on a case-by-case basis. In contrast, the numbers of researchers accessing open datasets on the internet where there are no DACs is much higher than through managed access systems [40]. Although it is anticipated that in the future there will be more information technology solutions, or e-governance tools, which will assist with the expedition of review and the minimization of unnecessary administrative burden electronically, currently this is not the case [48]. Another suggestion for cutting down on administration is “to implement an annual certification process, which would grant the certified researcher unrestricted access to study results with the condition that the data could only be used for research goals that do not compromise the participants’ privacy” [49]. To make such a system effective, resources would have to be put in to the development of effective tracking systems that would enable all of the uses of data to be monitored so that inappropriate uses of the data could be sanctioned.

## Moving forward

Genomics has been a leader in the sharing of data, because funders and researchers have endorsed the principle of open access and put considerable resources and expertise into the development of data sharing governance systems. To reach this point has required commitment to the curation and archiving of, and access to, datasets and the development of new ways of working, which are now an intrinsic part of the governance design and management structures for new research. Leading projects have set the standards and developed the systems that have been adopted by later research projects. However, these have been applied to individual projects, without a critical appraisal of the implications for the field as a whole. This incremental build-up of oversight systems may affect the long-term sustainability of data sharing. Commentators have argued that managed access datasets, which have become the norm in genomics, have a chilling effect on access requests to data. It is not clear whether this is because of the type of data that is held in managed access datasets, which may not be suitable for a broad range of scientific uses, or whether it is because governance requirements are too restrictive.

As descibed earlier, the difficulties of obtaining consent for future, unforeseen uses and the resources needed to go back for re-consent, if the original consent is inadequate, are a significant stumbling block to streamlined lawful and ethical data sharing. The current paper-based, up-front systems of obtaining consent are inadequate for the flows of data that are required for expedited data sharing. In the current system, once consent is obtained, research participants, who are the subjects of the study and take privacy risks, no longer have any control over the use of their data. In contrast, the dynamic consent approach developed as part of the ENCoRe project [50] uses information technology to provide a way in which research participants can become more engaged in how their personal information is used in research. This approach, which can transcend national boundaries, enables engagement with participants to be more streamlined and effective [34].

The ways in which genomics data sharing policies have been designed, and the strengths and weaknesses of the incremental approach described earlier, give rise to some important lessons for future policy development, both in genomics and in other fields of scientific endeavour in terms of sustainability (Box 1). It is clear that policies need to have a normative foundation to ensure that robust and coherent governance mechanisms are in place, not just in large projects but also in smaller ones. Therefore, it is essential that appropriate ethical and legal support and expertise is available for those who are developing the initial policies, as these become the templates for further studies. This is also essential for the design of meta-governance structures. In this respect, advice from experts on the ground as well as the use of a broader policy-making infrastructure will be essential to develop global meta-governance structures. One such policy-making infrastructure is ELSI 2.0, which is an initiative designed to enable a wide range of stakeholders to participate in global research and policy activities [51].

The administrative burden of access is a key challenge, not only for those developing and implementing policies, but also for researchers who wish to either share or access data. To address this challenge, better systems are needed so that access procedures and oversight mechanisms are proportionate to the risks involved for different types of data. In considering the proportionality of oversight, funders are ideally placed to drive the implementation of a system that minimizes administrative burden while providing appropriate protection. In the longer term, meta-level, global governance systems need to be developed, which incorporate information technology or e-governance solutions that can reward new types of contributions and cut down on unnecessary administration.

With the launch of the Global Alliance that seeks to build an infrastructure to share data with many researchers across the world [2], and with many funders considering how best to promote global data sharing, it is an opportune time to reflect on how data sharing policy has been developed and implemented. To make data sharing more efficient, economic and effective at a global level requires the development of a strategy that involves many stakeholders. This strategy cannot be nationally based but must be orientated to the needs of global research and be facilitated by global infrastructure. Access requirements need to be organized around classes of data rather than being organized around projects and studies. New web technologies need to be used to deal with the complex issues around recruitment, consent and citizen engagement. Incentives and rewards for data generation and sharing need to be linked with institutional career paths for researchers, particularly in academia. Greater attention and support needs to be given to the importance of incremental development of practice as well as to the administrative load that data sharing policy requires. The potential to use information technologies to track the use of data and ensure compliance with data sharing requirements still needs to be explored to develop global data sharing.

For data sharing to persist as a key element of genomics research, it is important that it be sustainable - that it continues effectively and durably into the future with appropriate oversight and protections for researchers and participants while still allowing research to proceed. Sustainability of data sharing is a complex issue and involves the sustainability of many different facets of the system: the data generation itself, the policies and procedures for sharing the data, the researchers who use the shared data, and the infrastructure that supports the enterprise [52]. Attention to the development and design of new governance structures at the project, consortia and global levels will go some way to ensuring that data continues to be accessed efficiently to achieve the aims of open access policies.

## Box 1. Key challenges and corresponding solutions for sustainability of data sharing

Challenges of developing data sharing governance

• Data sharing is often required as a condition of funding but funders have not prescribed how this should be done. This has resulted in a learning curve for researchers who have had to expend resources to archive datasets or, in the case of consortia, develop policy and governance systems to oversee access to datasets.

• For consortia, developing policy and governance systems to share data are costly and time-consuming to establish, so the first projects often become templates for the projects that follow.

• Current data access procedures and governance work well for individual projects, but their cumulative effect may result in access procedures becoming cumbersome and disproportionate to the risks associated with using some datasets.

• Separate and uncoordinated approval systems to access project data are at risk of becoming unduly bureaucratic and undermining research that requires data from a number of sources.

• New ways to reward data generators, such as through researcher IDs and the citation of resources in publications are in the process of development but still need to be uniformly recognized and implemented.

Solutions to ensure sustainability of data sharing

• Further research needs to be undertaken to understand the reasons for the sizeable differences in access rates between open access models and managed access models for data sharing.

• There needs to be appropriate ethical and legal support and expertise for those who are developing initial policies as these become the templates for further studies. Funders need to ensure that large projects have this support.

• Greater attention and appropriate funding needs to be given to the cost associated with managing data sharing activities.

• Better systems of triaging need to be developed to ensure that appropriate access procedures and oversight mechanisms are proportionate to the risks involved for access to different types of data.

• To ensure that access application systems are appropriately harmonized across the globe, funders could take the lead, informed by policy developed through the ELSI 2.0 infrastructure, in implementing a system that is more proportionate for lower risk data.

• Accreditation and researcher IDs could be used as part of an electronic meta-governance system to promote international research.

• Further work is needed to develop meta-governance structures at a global level that are accountable, transparent and representative of all stakeholders’ interests, but will also minimize bureaucracy and facilitate research.

## Abbreviations

DAC: Data access committee; dbGaP: Database of Genotypes and Phenotypes; EGA: European Genome-phenome Archive; ELSI: Ethical, legal and social implications; GWAS: Genome-wide association study; HGP: Human Genome Project; ID: Identifier; NIH: National Institutes of Health; PI: Principal investigator; WTCCC: Wellcome Trust Case Control Consortium.

## Competing interests

The authors declare that they have no competing interests.
